# Real-world evidence for steroidal mineralocorticoid receptor antagonists in patients with chronic kidney disease

**DOI:** 10.1007/s40620-022-01492-w

**Published:** 2022-11-23

**Authors:** Kerstin Folkerts, Aurelie Millier, Beata Smela, Elzbieta Olewinska, Niklas Schmedt, Paul Mernagh, Csaba P. Kovesdy

**Affiliations:** 1 Division of Nephrology, Department of Medicine, University of Tennessee Health Science Center, Memphis, TN USA; 2 Bayer AG, Wuppertal, Germany; 3 Creativ-Ceutical, Paris, France; 4 Creativ-Ceutical, Cracow, Poland; 5 Bayer AG, Berlin, Germany

**Keywords:** Chronic kidney disease, Type 2 diabetes, Mineralocorticoid receptor antagonists, Real world evidence

## Abstract

**Background:**

Mineralocorticoid receptor antagonists (MRAs) were shown to delay chronic kidney disease (CKD) progression in patients with hypertension and/or heart failure (HF) and proteinuria.

**Objective:**

We conducted a systematic literature review on real-world evidence to identify the literature gaps related to the efficacy and safety outcomes of MRAs administered to CKD patients.

**Results:**

A total of 751 records were identified of which, 23 studies (26 publications) were analyzed. Studies included heterogeneous populations, including the overall CKD, CKD and diabetes, CKD and HF, and CKD and a history of cardiovascular disease. Most of the studies were small and non-rigorous, resulting in a notable lack of evidence in these populations. In the overall CKD population, steroidal MRAs resulted in a significant or sustained eGFR reduction but no efficacy in delaying progression to end-stage kidney disease. No cardiovascular protection was found. Results for all-cause mortality and hospitalization for HF were inconsistent; however, the longest follow-up studies indicate similar or lower incidence for spironolactone non-users. Most results consistently reported a higher incidence of hyperkalemia among patients on steroidal MRAs in all CKD stages, and side effects led to high discontinuation rates in the real-world setting.

**Conclusions:**

Despite the limited availability of evidence on the effectiveness and safety of steroidal MRAs in CKD patients and subgroups with diabetes, HF or history of cardiovascular disease, MRAs were shown to have a limited effect on renal and cardiovascular outcomes. Gaps in the evidence regarding the efficacy and safety of MRAs are particularly relevant in diabetic CKD patients; therefore, further research is warranted.

**Supplementary Information:**

The online version contains supplementary material available at 10.1007/s40620-022-01492-w.

## Introduction

Chronic kidney disease (CKD) is a progressive and irreversible disease, causing gradual loss of kidney function. CKD is defined as the presence of abnormalities in kidney structure or function, occurring for longer than three months. Diagnosis is based on decreased glomerular filtration rate (GFR) and markers of kidney damage, usually persistent albuminuria or other structural abnormalities [1]. Comorbidities, including diabetes, hypertension and cardiovascular diseases are frequent in patients with CKD [2]. One of the key risk factors for the development of CKD is type 2 diabetes (T2D). Long-term hyperglycemia can be associated with serious complications such as retinopathy, neuropathy and nephropathy, which can lead to CKD [3].

Treatment of patients with CKD focuses on slowing the progression of the disease, reducing the risk of cardiovascular (CV) events and mortality, preventing end-stage kidney disease (ESKD), and managing the various metabolic and other complications of CKD [1, 3–5]. The key to slowing the progression of CKD is to treat all underlying conditions and to use disease-modifying drugs [5]. Patients with CKD are treated with angiotensin-converting enzyme inhibitors (ACEIs) or angiotensin receptor blockers (ARBs) to reduce CV risk, but benefits of other medications have been reported [6]. In patients with T2D, sodium/glucose cotransporter-2 inhibitors were found to protect the heart and kidney in addition to reducing blood glucose [7]. Both dapaglifozin (Farxiga, Astra Zeneca, Luton, UK) [8] and empagliflozin (Jardiance, Boehringer Ingelheim, Bracknell, UK) [9] reduce the risk of CKD progression and CV mortality [9, 10].

Mineralocorticoid receptor antagonists (MRAs) are primarily included in the standard management of heart failure (HF) and hypertension, yet they have been shown to delay the progression of CKD in patients with hypertension and/or HF and proteinuria [11–13]. Although the use of steroidal MRAs is low, it increases with disease severity, indicating that their use may be associated with greater disease burden. Moreover, patients receiving steroidal MRAs were more likely to have HF (at least threefold), other comorbidities, advanced CKD, and higher medication load [14]. The use of MRAs in patients with heart failure with reduced ejection fraction (HFrEF), resistant hypertension, and edema carries the risk of occurrence of adverse effects such as hyperkalemia, acute kidney injury, and gynecomastia when compared to placebo or standard of care. In a recent systematic literature review, Chung et al. reported that MRAs had uncertain effects on kidney failure, CV events and death. The study concluded that the identified evidence for the primary efficacy and safety outcomes was of very low to low certainty, mainly due to methodological limitations in the included studies, as well as serious imprecision in the estimated treatment. Finally, long-term data for MRAs on major patient-centered outcomes of interest were absent or sparse and most studies focused on surrogate outcomes [15].

Chung et al. [15] analyzed randomized controlled trials (RCTs) and quasi-RCTs with most studies lasting between 3 and 12 months. Complementing their review with a summary of results observed in real-world practice may provide more insight into such long-term outcomes. Therefore, the objective of this study was to conduct a systematic literature review on real-world evidence in patients with CKD treated with MRAs, to identify the gaps in the literature.

## Methods

### Literature search

This systematic literature review was performed based on a prespecified protocol and conducted following the methods developed by the Cochrane Handbook for Systematic Review of Interventions [16] and the Centre for Reviews and Dissemination [17] to reduce the risk of bias.

We searched Embase, Medline and Medline In-Process Citations, Daily Update and Epub Ahead of Print (access via the OVID interface) until October 2021. No language or geographical scope restrictions were imposed. Abstracts published before 2017 were excluded. Additionally, clinical trial registries (ClinicalTrials.gov, WHO International Clinical Trials Registry Platform, EU Clinical Trials Register, and PharmNet.Bund) were searched to identify ongoing and completed clinical trials. Selected conference websites were searched manually to make sure that all important data, even those published as abstracts only, were identified. The search was based on keywords and medical subject headings related to the population (chronic kidney disease, chronic kidney diseases, CKD, chronic nephropathy, chronic renal disease, chronic renal diseases, diabetic nephropathy, diabetic kidney disease, diabetic renal disease, renal insufficiency), MRAs (mineralocorticoid antagonist, aldosterone antagonist, mineralocorticoid receptor antagonism, mineralocorticoid receptor blockade) and individual drug names, as well as terms characteristic of observational studies. Abstracts and full texts were screened by two independent reviewers to select relevant articles based on the inclusion criteria. Any discrepancies between reviewers were resolved by a third reviewer. Afterwards, we grouped papers reporting results from the same studies.

We included all observational studies assessing the effectiveness and/or safety of MRAs in adult patients with CKD. We selected studies that reported at least one of the following effectiveness outcomes (CV events, renal events, including composites, and death), or safety outcomes (adverse events, including but not limited to hyperkalemia), or other outcomes such as persistence of treatment or treatment discontinuation. The scope of the review was intentionally broad to ensure high sensitivity. We excluded trials with fewer than 100 participants, as well as literature reviews, pooled analyses, editorials, letters, opinion articles, pre-clinical studies and general discussions. Studies with mixed populations of patients, including patients with ESKD or pediatric patients, were included in the review if they reported separate data for eligible patients.

### Data extraction process

Data from studies meeting the PICOS criteria were extracted using extraction templates created in Excel (Microsoft Corp., Redmond, WA, USA). Data were extracted by one reviewer, while another reviewer validated the accuracy of the extracted data. Data extraction included: publication details, study design, baseline characteristics, relevant results, and data necessary for quality assessment.

### Quality assessment

The Newcastle–Ottawa Scale was used to assess the methodological quality of the included real-world evidence (RWE) studies [18]. This tool uses a star rating system to assess the appropriateness of the three domains: selection of study groups, comparability of groups and ascertainment of exposure and outcomes for case–control and cohort studies. A star system is used to rate quality: the highest-quality studies are awarded a maximum of one star for each item except for an item related to comparability that allows the assignment of two stars. The total score ranges between zero up to nine stars.

## Results

A total of 751 records were identified from the literature search. After identifying duplicates and screening abstracts, 105 publications were reviewed as full texts. Finally, we considered 23 studies (26 publications), including 11 (11 publications) reporting results in the overall CKD population, three studies (four publications) in CKD in diabetes of which two reported results for CKD in T2D, 11 studies (11 publications) in CKD and HF (or reporting results on CKD with HF as a subgroup) and one study (one publication) in CKD and a history of cardiovascular disease (CVD) (reporting results on CKD with CVD history as a subgroup).

Several studies reported results for different populations. For example, Blankenburg et al. [14] presented results for overall CKD, CKD in T2D, as well as CKD (with or without T2D) and HF populations separately, and Tseng et al. [19] reported results for overall CKD, CKD and HF, as well as for CKD and history of CVD. In addition, Qu et al. [20] reported results for overall CKD and CKD in diabetes.

Of the included studies, 83% were retrospective based on many sources of data, including various medical registries or national and commercial hospital/clinical registries. Study cohorts of patients treated with MRAs varied from 105 [21] to 13,726 patients [22]. The duration of the follow-up ranged from one year [22–24] to a maximum of eight years [25].

The overall quality of the studies assessed with the Newcastle–Ottawa Scale ranged from poor to good. Case–control studies received from four to eight stars; one had good and two had fair quality. Cohort studies received from five to nine stars; 13 had good quality, two had fair quality and eight had poor quality. Caveats regarding quality included lack of information regarding adequacy of the follow up, no evidence that the outcome of interest was not present at start of the study, and lack of comparability of cohorts on the basis of the design or analysis. In two studies, published as abstracts only, information was not sufficient to make an assessment.

**Fig. 1:**
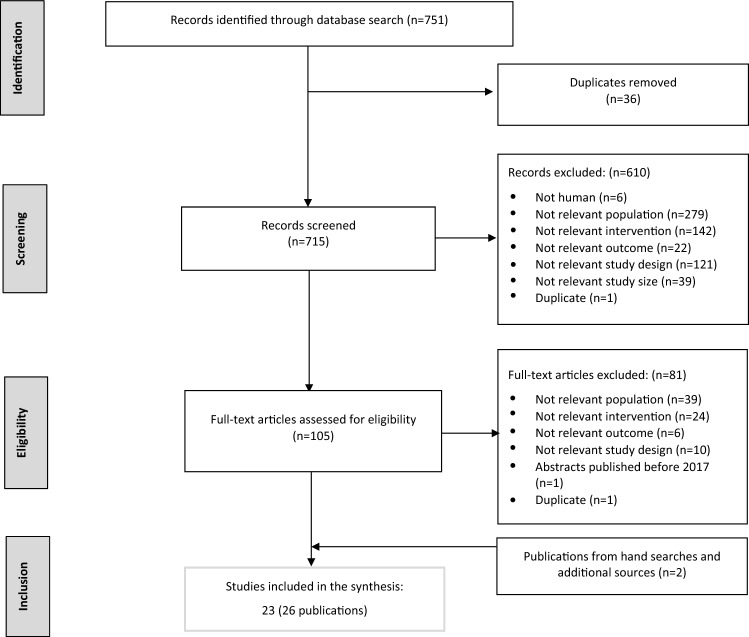
Summary of study flow

The study selection process is depicted in Fig. 1, and the characteristics of the included studies are described in Supplementary Table 1.

### RWE in overall CKD

Effectiveness and safety outcomes among patients with overall CKD treated with MRAs were presented in 11 studies (Table 1). The definition of the overall CKD population varied among the included studies (Supplementary Table 1); most were based on eGFR values, and frequently used a threshold of eGFR < 60 mL/min/1.73 m^2^. To illustrate this heterogeneity, one study[19] included patients with CKD in pre-dialysis stage 5, another included patients with CKD stage 3–4 [26], while another one was comprised of patients with CKD stage 2 or worse [27].

**Table 1:** Effectiveness and safety outcomes reported in the included studies

Author and year	Country	N*	Composite outcome	Renal outcomes				CV outcomes					All-cause mortality	All-cause hospitalisation	Persistence	Discontinuation	Safety		
				eGFR slope	Decrease in eGFR	Onset of ESKD	Doubling of serum Cr	CV death	HHF	HF	MI	Stroke					Hyperkalaemia	Hosp. due to hyperkalaemia	Worsening of renal function
*Overall CKD*																			
Blankenburg, et al. [30]	Germany	11,554 (5777)							✓	✓							✓		
Gay, et al. [49]	USA	235,032 (5889)													✓				
Qu, et al. [20]	China	560 (200)											✓	✓		✓	✓		
Blankenburg, Fett [14]	USA	229,004 (5899)								✓	✓	✓			✓	✓	✓		
Jun 2019^11^	Australia	20,184 (1648)															✓		
Trevisan, de Deco [22]	Sweden	13,726 (3788)^														✓	✓		
Linde, McEwan [52]	UK	100,572 (9687)														✓			
Yang, Kor [26]	Taiwan	14,669 (785)	✓			✓		✓	✓				✓					✓	
Gillis, Lees [28]	UK	7766 (402)		✓		✓											✓		
Tseng, Liu [19]	Taiwan	27,213 (1363)	✓					✓	✓				✓				✓	✓	
Hassan, Qureshi [27]	USA	850 (425)							✓				✓				✓		
Herget-Rosenthal, Dehnen [29]	Germany	803 (204)^				✓													
Surabenjawong 2013^1^	Thailand	534 (44)^															✓		
*CKD in diabetes*																			
Qu, et al. [20]	China	560 (200)											✓	✓					
Blankenburg, et al. [32]	USA	10,930 (5465)								✓		✓		✓	✓		✓		
Blankenburg, Fett [14]	USA	229,004 (5899)								✓	✓	✓			✓	✓	✓		
Blankenburg, Kovesdy [33]	USA	10,930 (5465)											✓						
Yamazaki, Yoshihara [25]	Japan	19,582 (2295)	✓														✓		
CKD and HF																			
Giannetti, et al. [50]	NR	1430 (314)														✓			
Buckallew, et al. [38]	USA	121 (121)															✓		
Mavrakanas, Giannetti [12]	NR	1,430 (314)	✓		✓		✓									✓			
Blankenburg, Fett [14]**	USA	229,004 (5899)								✓	✓	✓			✓	✓	✓		
Martinez-Milla, Garcia [36]	Spain	390 (156)	✓										✓						
Trevisan, de Deco [22]**	Sweden	13,726 (3788)^														✓			
Lofman, Szummer [35]	Sweden	45,071 (4470)											✓						
Cooper 2017^8^	USA	10,443 (446)^															✓		✓
Devesa, Cortes Garcia [34]	Spain	802 (390)^	✓										✓						
Trevisan, de Deco [22]**	Sweden	13,726 (3788)^															✓		
Tseng, Liu [19]**	Taiwan	27,213 (1363)							✓				✓						
Oh, Kang [21]	Korea	1035 (105)											✓	✓			✓		
Inampudi, Parvataneni [24]	USA	1140	✓						✓				✓	✓					
Lin, Yu [53]	Taiwan	801 (430)^											✓						
CKD and CVD history																			
Tseng, Liu [19]**	Taiwan	27,213 (1363)							✓				✓						

*CKD* chronic kidney disease, *Cr* creatinine, *CV* cardiovascular, CVD cardiovascular disease, *eGFR* estimated glomerular filtration rate, *ESKD* end stage kidney disease, *Hosp*. hospitalization, *HF* heart failure, *HHF* hospitalization for heart failure, *MI* myocardial infarction, *N* sample size, *UK* United Kingdom, *USA* United States of America

*Data presented as the total number of patients included (total number of patients treated with MRAs), unless otherwise indicated

**Results for subgroup of patients with HF

^Total (CKD)

✓Outcome reported in study

### Effectiveness

#### Renal outcomes

The results are inconclusive when it comes to eGFR decline. On one hand, in the Gillis et al. study [28], patients with CKD treated with MRAs had a significantly greater decline in eGFR per year compared to the non-MRA treatment arm at the end of the follow up (3 vs. 2.3 mL/min/1.73 m^2^, p < 0.001). On the other hand, in the Herget-Rosenthal et al. study [29], the odds ratio of eGFR decline > 7.5 mL/min/1.73 m^2^ among patients at high risk of CKD exclusively managed in primary care, where they were treated with MRAs, was 0.9 (95% CI: [0.12; 1.36]), compared to patients not receiving MRAs.

The results are inconclusive regarding the progression to ESKD as well. Gillis et al. [28] reported a comparable proportion of patients progressing to ESKD for MRA users and MRA non-users (11.4% vs. 14.9%, p = 0.06). On the contrary, Yang et al. [26] reported a lower cumulative incidence of ESKD for spironolactone users in comparison to those not taking spironolactone (p < 0.001).

#### CV outcomes

The event rates of major adverse cardiovascular events, defined as myocardial infarction or ischemic stroke, were found to be similar between MRA users vs. non-users. Yang et al. reported that event rates in spironolactone users and non-users were 24.94 vs. 24.83/1000 person-years among patients with CKD stage 3–4 [26], and Tseng et al. reported 3.2 vs. 2.2/100 person-years in pre-dialysis patients in CKD stage 5 [19].

No significant difference in CV death rates was identified between spironolactone users and non-users, either in patients with CKD stage 3–4 (11.41 vs. 10.97/ 1000 person-years[26], respectively) or in patients in pre-dialysis CKD stage 5 (1.4 vs. 0.7/person-years[19], respectively), after adjustment was considered in the statistical analysis.

The incidence of hospitalization for HF was not significantly different between spironolactone users and non-users, as reported by Yang et al. (event rates of 12.92 vs. 15.34/1000 person-years in patients with CKD stage 3–4 [26], p = 0.225). Blankenburg et al. [30] reported higher event rates among steroidal MRA users than in non-users (0.704 vs 0.394 per person-year). In the Hassan et al. study [27], the hazard ratio for hospitalization for HF was found to be lower in patients treated with spironolactone than in the controls (HR = 0.37, 95% CI [0.19; 0.74], p < 0.0001).

Blankenburg et al. [30] found that steroidal MRA users had almost twice the frequency of HF compared to patients not treated with MRAs (83.0% vs 46.8%). In another study by Blankenburg et al. [14], the incidence of HF and myocardial infarction was numerically higher among patients treated with spironolactone (for more and less than six months) compared to the non-MRA treatment arm, however, no statistical tests were performed. The percentage of patients that experienced strokes was similar among patients treated with spironolactone for more than six months and non-MRA users (p-value not reported).

#### All-cause mortality

In the Tseng et al. study [19], over a median follow-up of 31 months (85,758 person-years), the use of spironolactone was associated with a significantly higher risk of all-cause mortality, irrespective of the drug dose (HR = 2.1, 95% CI [1.93; 2.27], p < 0.001). In the Hassan, et al. study [27], a numerically greater risk of all-cause mortality was reported for MRA users, compared to non-MRA users; however, results were not statistically significant. In the Yang et al. study [26], the difference between the incidence of all-cause death in spironolactone users versus non-users was not significant (event rates 64.42 vs. 60.47/1000 person-years, p = 0.432). In the Qu et al. [20] study, spironolactone use was related to significantly lower risk of all-cause mortality compared to standard treatment (HR = 0.389, 95% CI [0.276; 0.548], p < 0.001). Moreover, the reduction in the risk of all-cause mortality in spironolactone users was consistent among analyzed subgroups, including patients with different levels of eGFR.

#### All-cause hospitalization

In the Qu et al. [20] study, spironolactone use was associated with significantly lower risk of re-hospitalization compared to standard treatment (HR = 0.664 95% CI [0.522; 0.846], p = 0.004). Additionally, the reduction in the risk of re-hospitalization among spironolactone users was consistent among analyzed subgroups including patients with different levels of eGFR.

The results for renal outcomes, CV outcomes, all-cause mortality and all-cause hospitalization are shown in Table 2.

**Table 2:** Effectiveness outcomes: overall CKD

Outcome	Author and name	Population	Timepoint	MRAs			Controls			HR [95% CI], p-value
				Treatment arm	N/n (%)	Event rate/1000 PY	Reference	N/n (%)	Event rate/1000 PY	
*Renal*										
Change in eGFR per year (mL/min/1.73 m^2^)*	Gillis, Lees [28]	Patients with CKD	NR	MRAs	-3‡	NR	Non-MRA users	-2.3‡	NR	P < 0.001
ESKD	Yang, Kor [26]	Patients with CKD stage 3–4	•Spironolactone users: 3.57 (3.2)‡ years •Spironolactone non-users: 3.24 (3.23)‡ years	Spironolactone	693/88♦	39.2	Non-Spironolactone users	1386/266♦	53.69	0.65 [0.51; 0.83]^Cru^, p < 0.001 0.66 [0.51; 0.84] ^A^, p < 0.001
	Gillis, Lees [28]	Patients with CKD	NR	MRAs	402/46 (11.4)	NR	Non-MRA users	7364/1097 (14.9)	NR	p = 0.06
	Herget-Rosenthal, Dehnen [29]	Patients with or at high risk of CKD exclusively managed in primary care	NR	Aldosterone antagonists	204	NR	NR	NR	NR	0.90 [0.12; 1.36]▲**
*Cardiovascular*										
*MACE* (composite of MI and IS)	Yang, Kor [26]	Patients with CKD stage 3–4	•Spironolactone users: 3.57 (3.2)‡ years •Spironolactone non-users: 3.24 (3.23)‡ years	Spironolactone	693/56♦^P^	24.94	Non-Spironolactone users	1386/123♦^P^	24.83	0.93 [0.68; 1.27]^Cru^, p = 0.647 0.93 [0.67; 1.28] ^A^, p = 0.647
	Tseng, Liu [19]	Patients with CKD in pre-dialysis stage 5	NR	Spironolactone	1363/82 (6.02)	3.2^	Non-Spironolactone users	25,850/1794 (6.94)	2.2^	1.40 [1.12; 1.75]^Cru^ 0.89 [0.71; 1.12]^A^ 0.89 [0.69; 1.14]^P^ p = 0.002
CV death	Yang, Kor [26]	Patients with CKD stage 3–4	•Spironolactone users: 3.57 (3.2)‡ years •Spironolactone non-users: 3.24 (3.23)‡ years	Spironolactone	693/34♦^P^	11.41	Non-Spironolactone users	1386/70♦^P^	10.97	1.02 [0.67; 1.53]^Cru^, p = 0.941 1.14 [0.75; 1.74]^A^, p = 0.533
	Tseng, Liu [19]	Patients with CKD in pre-dialysis stage 5	Within 120 days◊	Spironolactone	1124/31 (2.76)	1.4^	Non-Spironolactone users	22,557/541 (2.4)	0.7^	1.79 [1.25; 2.58]^Cru^ 0.71 [0.48; 1.03]^A^
HHF	Yang, Kor [26]	Patients with CKD stage 3–4	•Spironolactone users: 3.57 (3.2)‡ years •Spironolactone non-users: 3.24 (3.23)‡ years	Spironolactone	693/29♦^P^	12.92	Non-Spironolactone users	1386/76♦^P^	15.34	0.77 [0.5; 1.19]^Cru^, p = 0.238 0.77 [0.5; 1.18]^A^, p = 0.225
	Blankenburg, et al. [30]	Adult CKD patients	Within 1 year	MRAs	11,554/5,777 (50)	0.704^$^	Non-MRAs	11,554/5,777 (50)	0.394^$^	
	Tseng, Liu [19]	Patients with CKD in pre-dialysis stage 5	Within 120 days◊	Spironolactone	1124/92 (8.19)	4.5^	Non-Spironolactone users	22,557/1001 (4.44)	1.4^	2.64 [2.13; 3.27]^Cru^ 1.37 [1.09; 1.72]^A^
	Hassan, Qureshi [27]	Adult ambulatory patients who were diagnosed with CKD stage 2 or worse	4.6 years (3.3; 5.8)†	Spironolactone (cases)	425/48 (11.3)	NR	Non-Spironolactone users	425/82 (19.3)	NR	0.37 [0.19; 0.74]^A^, p = 0.005
HF	Blankenburg, Fett [14]	Patients with CKD	< 6 months	Spironolactone	869/99 (11.4)	NR	Non-MRA users	112,730/3,066 (2.7)	NR	NR
			≥ 6 months		481/30 (6.2)	NR		112,730/3,066 (2.7)	NR	NR
	Blankenburg, et al. [30]	Adult CKD patients	Within 1 year	MRAs	11,554/5,777 (50)	83%	Non-MRAs	11,554/5,777 (50)	46.8%	NR
MI	Blankenburg, Fett [14]	Patients with CKD	< 6 months	Spironolactone	869/59 (6.8)	NR	Non-MRA users	112,730/3440 (3.1)	NR	NR
			≥ 6 months		481/26 (5.4)	NR		112,730/3440 (3.1)	NR	NR
Stroke	Blankenburg, Fett [14]	Patients with CKD	< 6 months	Spironolactone	869/120 (13.8)	NR	Non-MRA users	112,730/9821 (8.7)	NR	NR
			≥ 6 months		481/42 (8.7)	NR		112,730/9821 (8.7)	NR	NR
*All-cause mortality*										
All-cause mortality	Qu, et al. [20]	Patients with AMI and CKD	30 months	Spironolactone group	41 (20.5)	NR	Standard treatment group	160 (44.4)	NR	0.389 [0.276; 0.548], p < 0.001
		eGFR 30- < 60 mL/min/1.73 m^2^	30 months	Spironolactone group	NR	NR	Standard treatment group	NR	NR	0.557 [0.363; 0.855], NR 0.167^
		eGFR 15- < 30 mL/min/1.73 m^2^	30 months	Spironolactone group	NR	NR	Standard treatment group	NR	NR	0.32 [0.152; 0.671], NR 0.167^
		eGFR < 15 mL/min/1.73 m^2^	30 months	Spironolactone group	NR	NR	Standard treatment group	NR	NR	0.322 [0.099; 1.051], NR 0.167^
Risk for all-cause mortality	Yang, Kor [26]	Patients with CKD stage 3–4	•Spironolactone users: 3.57 (3.2)‡ years •Spironolactone non-users: 3.24 (3.23)‡ years	Spironolactone	693/192♦^P^	64.42	Non-Spironolactone users	1386/386♦^P^	60.47	1.07 [0.9; 1.27]^Cru^, p = 432 1.1 [0.92; 1.3]^A^, p = 0.294
All-cause mortality	Tseng, Liu [19]	Patients with CKD in pre-dialysis stage 5	Within 120 days	Spironolactone	1124/530 (47.15)	23.5^	Non-Spironolactone users	22,557/7582 (33.61)	10.2^	2.11 [1.94; 2.31]^Cru^ 1.06 [0.96; 1.16]^A^
Risks for all-cause mortality		Patients with CKD in pre-dialysis stage 5	NR	Spironolactone	1363/654 (47.98)	24.7^	Non-Spironolactone users	25,850/8801 (34.05)	10.6^	2.1 [1.93; 2.27]^Cru^, p < 0.001 1.35 [1.24; 1.46] ^A^ 1.39 [1.26; 1.53]^P^
Reduction in all-cause mortality	Hassan, Qureshi [27]	Adult ambulatory patients who were diagnosed with CKD stage 2 or worse	4.6 years (3.3; 5.8)†	Spironolactone (cases)	425/NR	NR	Control group	425/NR	NR	0.87 [0.66; 1.13]^A^, p = 0.29***
*All-cause hospitalization*										
Re-hospitalization	Qu, et al. [20]	Patients with AMI and CKD	30 months	Spironolactone group	95 (47.5)	NR	Standard treatment group	217 (60.3)	NR	0.664 [0.522; 0.846], p = 0.004
		eGFR 30- < 60 mL/min/1.73 m^2^	30 months	Spironolactone group	NR	NR	Standard treatment group	NR	NR	0.897 [0.666; 1.207], NR 0.075^
		eGFR 15- < 30 mL/min/1.73 m^2^	30 months	Spironolactone group	NR	NR	Standard treatment group	NR	NR	0.612 [0.36; 1.04], NR 0.075^
		eGFR < 15 mL/min/1.73 m^2^	30 months	Spironolactone group	NR	NR	Standard treatment group	NR	NR	0.547 [0.215; 1.388], NR 0.075^

*A* adjusted, *AMI* acute myocardial infarction, *CI* confidence interval, *CKD* chronic kidney disease, *Cru* crude analysis, *CV* cardiovascular, *eGFR* estimated glomerular rate, *ESKD* end stage kidney disease, *HF* heart failure, *HHF* hospitalization for heart failure, *HR* hazard ratio, *IS* ischemic stroke, *MACE* major adverse cardiac event, *MI* myocardial infarction, *MRAs* mineralocorticoid receptor antagonists, *N*/*n* number of patients/number of patients with an event, *NR* not reported, *P* after propensity score matching, *PY* person-years

*Available results for eplerenone, spironolactone, low dose MRA and high dose MRA (not presented here)

**Risk factors associated with eGFR decline > 7.5 mL/min/1.73 m^2^

***Reduction in all-cause-mortality; number of deaths 850/369 (43.41)

♦Number of events

^Per 100 person-years

$Per 1000 person-years

†Median (IQR)

‡Mean (SD)

▲Odds ratio

◊Available also data for 30 and 60 days

^p for interaction (eGFR 30–< 60 mL/min/1.73 m^2^ vs eGFR 15–< 30 mL/min/1.73 m^2^ vs eGFR < 15 mL/min/1.73 m^2^)

### Safety

The most frequently reported safety outcomes were hyperkalemia and hospitalization due to hyperkalemia (Table 3). The definition of hyperkalemia differed greatly among studies and included risk of any, mild or moderate to severe hyperkalemia, incident hyperkalemia and various serum potassium levels.

**Table 3:** Results on hyperkalemia: overall CKD

Author and name	Outcome definition	Population	Timepoint	MRAs			Controls			HR [95% CI], p-value
				Treatment arm	N/n (%)	Event rate/1000 PY	Reference	N/n (%)	Event rate/1000 PY	
*Hyperkalemia*										
Qu, et al. [20]	Serum potassium levels collected during subsequent FU more than 5.5 mmol/L	Patients with AMI and CKD	30 months	Spironolactone group	18 (9)	NR	Standard treatment group	18 (5)	NR	1.879 [0.954; 3.7], p = 0.068
		CKD stage 3	30 months	Spironolactone group	19 (5)	NR	Standard treatment group	NR	NR	NR, NR 0.133^
		CKD stage 4	30 months	Spironolactone group	12 (9.8)	NR	Standard treatment group	NR	NR	NR, NR 0.133^
		CKD stage 5	30 months	Spironolactone group	5 (8.5)	NR	Standard treatment group	NR	NR	NR, NR 0.133^
Blankenburg, et al. [30]	NR	Adult CKD patients	Within 1 year	MRA	11,554/5,777 (50)	105	Non-MRA	11,554/5,777 (50)	38	NR
Blankenburg, Fett [14]	NR	Patients with CKD	< 6 months	Spironolactone	869/94 (10.8)	NR	Non-MRA users	112,730/5187 (4.6)	NR	NR
			≥ 6 months		481/53 (11)	NR				NR
Blankenburg, Fett [14]	NR	Patients with CKD	< 6 months	Spironolactone	512/78 (15.2)	NR	Non-MRA users	14,653/1593 (10.9)	NR	NR
			≥ 6 months		373/53 (14.2)	NR				NR
Jun, Jardine [31]	Serum potassium > 6 mmol/L or coded or free-text recorded diagnosis of hyperkalemia	Patients with CKD who were prescribed an RAAS	NR	Aldosterone antagonist (eplerenone, spironolactone)	1648/NR	NR	ACEI	NR	NR	1.5 [1.29; 1.75]^U^, p < 0.001 1.53 [1.25; 1.87]^F^, p < 0.001
Trevisan, de Deco [22]	Predictors of any hyperkalemia risk (> 5.0 mmol/L)	Patients with CKD and eGFR 45–60 mL/min/1.73 m^2^	At baseline (FU: 1 year)	MRA users (spironolactone, eplerenone)	NR	NR	No CKD (eGFR > 60 mL/min/1.73m^2^)	NR	NR	1.49 [1.34; 1.65]
		Patients with CKD and eGFR 30–45 mL/min/1.73 m^2^								2.08 [1.84; 2.33]
		Patients with CKD and eGFR < 30 mL/min/1.73 m^2^								2.51 [2.09; 3.02]
	Predictors of mild hyperkalemia risk (> 5.0–5.5 mmol/L)	Patients with CKD and eGFR 45–60 mL/min/1.73 m^2^	At baseline (FU: 1 year)	MRA users (spironolactone, eplerenone)	NR	NR	No CKD (eGFR > 60 mL/min/1.73m^2^)	NR	NR	1.45 [1.29; 1.62]
		Patients with CKD and eGFR 30–45 mL/min/1.73 m^2^								1.89 [1.66; 2.16]
		Patients with CKD and eGFR < 30 mL/min/1.73 m^2^								1.96 [1.58; 2.44]
	Predictors of moderate/severe hyperkalemia risk (> 5.5 mmol/L)	Patients with CKD and eGFR 45–60 mL/min/1.73 m^2^	At baseline (FU: 1 year)	MRA users (spironolactone, eplerenone)	NR	NR	No CKD (eGFR > 60 mL/min/1.73m^2^)	NR	NR	1.73 [1.46; 2.04]
		Patients with CKD and eGFR 30–45 mL/min/1.73 m^2^								2.61 [2.18; 3.14]
		Patients with CKD and eGFR < 30 mL/min/1.73 m^2^								4.03 [3.12; 5.19]
	Any hyperkalemia risk (K+ > 5.0 mmol/L)	Patients with CKD and eGFR 45–60 mL/min/1.73 m^2^	At baseline (FU: 1 year)	MRA users (spironolactone, eplerenone)	NR	NR	No CKD (eGFR > 60 mL/min/1.73m^2^)	NR	NR	1.46 [1.31; 1.63]
		Patients with CKD and eGFR 30–45 mL/min/1.73 m^2^								2.17 [1.92; 2.47]
		Patients with CKD and eGFR < 30 mL/min/1.73 m^2^								2.78 [2.29; 3.36]
	Mild hyperkalemia risk (K+ > 5.0–5.5 mmol/L)	Patients with CKD and eGFR 45–60 mL/min/1.73 m^2^	At baseline (FU: 1 year)	MRA users (spironolactone, eplerenone)	NR	NR	No CKD (eGFR > 60 mL/min/1.73m^2^)	NR	NR	1.36 [1.2; 1.54]
		Patients with CKD and eGFR 30–45 mL/min/1.73 m^2^								1.95 [1.69; 2.25]
		Patients with CKD and eGFR < 30 mL/min/1.73 m^2^								2.13 [1.69; 2.68]
	Moderate/severe hyperkalemia risk (K+ > 5.5 mmol/L.)	Patients with CKD and eGFR 45–60 mL/min/1.73 m^2^	At baseline (FU: 1 year)	MRA users (spironolactone, eplerenone)	NR	NR	No CKD (eGFR > 60 mL/min/1.73m^2^)	NR	NR	1.8 [1.5; 2.15]
		Patients with CKD and eGFR 30–45 mL/min/1.73 m^2^								2.87 [2.36; 3.5]
		Patients with CKD and eGFR < 30 mL/min/1.73 m^2^								4.64 [3.56; 6.05]
Gillis, Lees [28]	Potassium > 6.5 mmol/L	Patients with CKD	NR	MRAs	402/68 (16.9)	NR	Non-MRA users	7364/1,141 (15.5)	NR	p = 0.60
Tseng, Liu [19]	NR	Patients with CKD in pre-dialysis stage 5	Within 90 days	Spironolactone (accumulated dose > 1750 mg)	NR	NR	Spironolactone non-users	NR	NR	1.08 [0.9; 1.3]^Cru^ 0.96 [0.8; 1.16]^A^ 0.96 [0.79; 1.17]^P^
				Spironolactone (accumulated dose ≤ 1750 mg)	NR	NR		NR	NR	0.98 [0.81; 1.19]^Cru^ 0.87 [0.72; 1.06]^A^ 0.87 [0.71; 1.07]^P^
				Spironolactone (mean daily dose > 12.5 mg)	NR	NR	Spironolactone non-users	NR	NR	0.95 [0.8; 1.12]^Cru^ 0.95 [0.8; 1.12]^A^ 0.94 [0.78; 1.12]^P^
				Spironolactone (mean daily dose ≤ 12.5 mg)	NR	NR		NR	NR	0.89 [0.72; 1.1]^Cru^ 0.89 [0.72; 1.1]^A^ 0.9 [0.72; 1.14]^P^
Hassan, Qureshi [27]	Mild hyperkalemia 5.5–6 mEq/L)	Patients with CKD, ambulatory, stage 2 or worse*	4.6 (3.3; 5.8)^+^ years	Spironolactone	402/20 (5)	NR	Spironolactone non-users	7364/295 (4)	NR	NR
	Severe hyperkalemia > 6 mEq/L)				402/12 (3)	NR		7364/147 (2)	NR	NR
Surabenjawong, Thunpiphat [23]	Serum potassium > 5.0 mmol/L	Patients with CKD taking combination of spironolactone and ACEIs or ARBs	NR	Spironolactone	44/9 (20.5)	NR	Non-hyperkalemia	NR	NR	2.37 [1.07; 5.22] ╦^Cru^, p = 0.03 2.47 [1.07; 5.7] ╦^A^, p = 0.03
Hospitalization for hyperkalemia										
Yang, Kor [26]	NR	Patients with CKD stage 3–4	3.57 (3.2)‡ years	Spironolactone	693/123 (17.75)	54.79	Spironolactone non-users	1386/92 (6.64)	18.57	2.98 [2.28; 3.9]^Cru^, p < 0.001 3.17 [2.41; 4.17]^A^, p < 0.001
Tseng, Liu [19]	NR	Patients with CKD in pre-dialysis stage 5	31 (14; 57)^+^ months	Spironolactone	1363/NR	9.8†	Spironolactone non-users	25,850/NR	7.1†	1156 ^OPY^ 1.04 [0.91; 1.19]^Cru^ 0.92 [0.81; 1.06]^A^ 0.93 [0.79; 1.07]^P^

*ARBs* angiotensin receptor blockers, *ACEI* angiotensin-converting-enzyme inhibitor, *AMI* acute myocardial infarction, *CI* confidence interval, *CKD* chronic kidney disease, *eGFR* estimated glomerular filtration rate, *FU* follow up, *HR* hazard ratio, *MRA* mineralocorticoid receptor antagonist, *N*/*n* number of patients/number of patients with an event, *NR* not reported, *PY* person-years, *RAAS* renin–angiotensin–aldosterone system

^p for interaction (CKD stage 3 vs CKD stage 4 vs CKD stage 5)

*As defined by the CKD-EPI equation

^+^Median (IQR)

‡Mean (SD)

╦Odds ratio

^A^Adjusted; ^Cru^ crude ^FU^ full multivariable model; ^OPY^ observed person-years; ^P^ propensity score matching; ^U^ univariate analysis

Around 30% of the studies did not use statistical tests to compare the rates between MRA users and non-users; however, the rates were generally higher for MRA users. Only four studies calculated p-values. Blankenburg et al. [30] reported a higher event rate in steroidal MRA users than in non-users (0.105 vs 0.038 per person-year). In the Jun et al. study [31], a likelihood of medication change following incident hyperkalemia was significantly higher in patients on MRAs (eplerenone or spironolactone) in comparison to ACEI treatment (HR = 1.5, 95% CI [1.29–1.75]; p < 0.001). Trevisan et al. [22] reported a significantly increased risk of hyperkalemia after initiation of MRA therapy in patients with CKD across all stages compared to patients without CKD. Gillis et al. [28] observed no significant difference (p = 0.60). In the Qu et al. [20] study, assessing CKD patients with acute myocardial infarction, hyperkalemia occurred in the spironolactone group more often than in the standard treatment group (9.0%, 5.0%, respectively, HR = 1.879, 95% CI [0.954; 3.700], p = 0.068). In the same study, hyperkalemia occurred in 5.0%, 9.8% and 8.5% in the CKD stage 3, 4 and 5 groups, respectively, however the differences among the CKD groups were not statistically significant (p < 0.133). In the study of Yang et al. [26], spironolactone use was associated with a significantly higher risk of hyperkalemia-related hospitalization in comparison to spironolactone non-use across all study subgroups and spironolactone doses (HR = 2.98, 95% CI [2.28; 3.9], p < 0.001) in patients with CKD stage 3–4. In the Tseng et al. study[19], no statistical difference between the incidence of hospitalization for hyperkalemia between spironolactone users and non-users was found (event rates 9.8 vs. 7.1/100 person-years, HR = 1.04, 95%CI [0.91; 1.19]). However, hyperkalemia-associated hospitalization was defined differently in those studies. Yang et al. [26] used the first listed ICD-9 code 428 or 276.7 at discharge, while Tseng et al. [19] used the code 276.7 at discharge or the administration of potassium-lowering agents.

### RWE in subgroups of CKD in diabetes (T2D and/or T1D)

The availability of evidence for CKD in T2D was poor: the results are from two retrospective studies (3 publications) assessing spironolactone [14, 32, 33] conducted in the USA. Blankenburg et al. [14] included adult patients with a first diagnosis of CKD and patients with a first prescription for steroidal MRAs, but results were presented also for a subgroup of patients with CKD and T2D which comprised of 1360 MRA users and 75,616 MRA non-users. Patients using spironolactone or eplerenone were searched; however, due to the low number of patients receiving eplerenone, only data from spironolactone users were reported as MRA users. In another paper by Blankenburg et al. [32, 33], matched cohorts of spironolactone users and non-users with CKD and T2D (n = 5465 each) were assessed.

Two other studies were conducted among patients regardless of the type of diabetes. The study conducted by Yamazaki et al. [25], published as an abstract, assessed MRAs in CKD in diabetes, yet, no details regarding the type of diabetes were reported. This study included 19,582 patients of whom 2295 were MRA users. Another study conducted by Qu et al. [20] in China and published as a manuscript, assessed spironolactone in CKD in diabetes. This study included 560 patients of whom 200 were spironolactone users.

### Effectiveness

#### Renal outcomes

No result was reported in the identified studies.

#### CV outcomes

In the study by Blankenburg et al. [14], the percentage of patients who experienced myocardial infarction, stroke and HF was numerically higher among patients treated with spironolactone compared to non-MRA users; yet, residual confounding could be a cause, while at the same time they had more comorbidities, consumed more medications, and used more healthcare resources. Also, as reported by Blankenburg et al. [32], patients who were not persistently on spironolactone presented with a higher incidence of stroke, ischemic stroke and HF events compared to persistent users (59.7% vs 43.4%, 31.3% vs 27.5% and 27.5% and 23.5%, respectively). In the population of patients with CKD and diabetes studied by Yamazaki et al., the risk of composite CV outcomes was significantly higher among the subgroup of patients with an eGFR of 30–44 mL/min/1.73 m^2^ compared to patients with an eGFR of 45–59 mL/min/1.73 m^2^ (HR = 1.22, 95% CI [1.09; 1.36]) [25].

#### All-cause mortality

Results reported by Blankenburg et al. [33] for the younger sub-cohort of patients with CKD and T2D with lower rates of HF showed that the mortality rate/person-years was 4% vs. 7% for spironolactone users and non-users, respectively. In the Qu et al. [20] study, an association with statistically significantly lower risk of all-cause mortality in the spironolactone users compared to standard treatment group was reported among the CKD in diabetes population (HR = 0.338 [0.184; 0.619], p for interaction (history of diabetes mellitus—Yes vs No): 0.544).

#### All-cause hospitalization

Blankenburg et al. [32] reported results on all-cause hospitalization for patients with CKD and T2D. In the overall population and across all stages of CKD, treatment with spironolactone was associated with numerically higher rates of all-cause hospitalization compared with those among non-spironolactone users.

In the Qu et al. [20] study, spironolactone use was associated with a numerically lower risk of re-hospitalization compared to standard treatment among CKD in the diabetes population, however the results were not statistically significant (HR = 0.699 [0.468; 1.046], p for interaction (history of diabetes mellitus—Yes vs No): 0.833).

### Safety

#### Hyperkalemia

Blankenburg et al. [14, 32] reported a numerically higher incidence of hyperkalemia among spironolactone users than among non-users. The rate was highest in patients on spironolactone for less than 6 months (16.7%) and was lower in patients treated with spironolactone for longer than 6 months (14.3%). In patients not using spironolactone, it was 9.1% [14]. Also, a higher incidence of hyperkalemia was observed among non-persistent patients compared to persistent patients (30.4% and 24.3%, respectively) [32]. Yamazaki et al. [25] reported a cumulative incidence of hyperkalemia among Japanese patients with CKD and diabetes treated with MRAs of 7.63%, but did not provide information about CKD stage and the characteristics of diabetes.

The results for effectiveness and safety in a subgroup of CKD in diabetes are shown in Table 4.

**Table 4:** Results on effectiveness outcomes: CKD in diabetes

Outcome	Author and name	Population	Timepoint	MRAs			Controls			HR [95% CI], p-value
				Treatment arm	N/n (%)	Event rate/1000 PY	Reference	N/n (%)	Event rate/1000 PY	
*CV outcome*										
MI	Blankenburg, Fett [14]	Patients with CKD and T2D	During FU*	Spironolactone < 6 months	891/105 (11.8)	NR	Non-MRA users	75,616/3975 (5.3)	NR	NR
				Spironolactone ≥ 6 months	469/35 (7.5)	NR	Non-MRA users	75,616/3975 (5.3)	NR	NR
HF	Blankenburg, Fett [14]	Patients with CKD and T2D	During FU*	Spironolactone < 6 months	891/203 (22.8)	NR	Non-MRA users	75,616/4,418 (5.8)	NR	NR
				Spironolactone ≥ 6 months	469/39 (8.3)	NR	Non-MRA users	75,616/4,418 (5.8)	NR	NR
	Blankenburg, et al. [32]	Patients with CKD and T2D	Within 60 days	Spironolactone	5,465/3,263 (59.7)	NR	Spironolactone non-users	5,465/2,372 (43.4)	NR	NR
				Non-persistent users	2,630/1,670 (63.5)					
				Persistent users	2,800/1,492 (53.3)					
Stroke	Blankenburg, Fett [14]	Patients with CKD and T2D	During FU*	Spironolactone < 6 months	891/179 (20.1)	NR	Non-MRA users	75,616/9,532 (12.6)	NR	NR
				Spironolactone ≥ 6 months	469/63 (13.4)	NR	Non-MRA users	75,616/9,532 (12.6)	NR	NR
Any stroke	Blankenburg, et al. [32]	Patients with CKD and T2D	Within 60 days	Spironolactone—total	5,465/1,711 (31.3)	NR	Spironolactone non-users	5,465/1,503 (27.5)	NR	NR
				Non-persistent users	2,630/839 (31.9)					
				Persistent users	2,800/739 (26.4)					
Ischemic stroke	Blankenburg, et al. [32]	Patients with CKD and T2D	Within 60 days	Spironolactone	5,465/1,711 (31.3)	NR	Spironolactone non-users	5,465/1,503 (27.5)	NR	NR
				Non-persistent users	2,630/655 (24.9)					
				Persistent users	2,800/619 (22.1)					
All-cause mortality										
All-cause mortality	Qu, et al. [20]	CKD in diabetes	30 months	Spironolactone group	NR	NR	Standard treatment group	NR	NR	0.338 [0.184; 0.619], NR 0.544^
Mortality rate/PY	Blankenburg, Kovesdy [33]	Patients with CKD and T2D (younger sub-cohort)	1 year	Spironolactone	1431/NR	4	Spironolactone non-users	1431/NR	7	NR
All-cause hospitalization										
Re-hospitalization	Qu, et al. [20]	CKD in diabetes	30 months	Spironolactone group	NR	NR	Standard treatment group	NR	NR	0.699 [0.468; 1.046], NR 0.833^
All-cause hospitalization	Blankenburg, et al. [32]	Patients with CKD and T2D	During FU	Spironolactone—total	5,465/3,405 (62.3)	NR	Spironolactone non-users	5,465/2,743 (50.2)	NR	NR
				Spironolactone – CKD 1	186/84 (44.9)			186/57 (30.8)		
				Spironolactone—CKD 2	497/229 (46.1)			497/190 (38.3)		
				Spironolactone – CKD 3	2,120/1,291 (60.9)			2,120/1,018 (48)		
				Spironolactone – CKD 4	372/279 (74.9)			372/228 (61.2)		
				Spironolactone – CKD 5/ESKD/RRT	650/494 (76)			650/428 (65.8)		
*Hyperkalemia*										
Hyperkalemia**	Blankenburg, Fett [14]	Patients with CKD and T2D	During FU*	Spironolactone < 6 months	891/149 (16.7)	NR	Non-MRA users	75,616/6,890 (9.1)	NR	NR
				Spironolactone ≥ 6 months	469/67 (14.3)	NR	Non-MRA users	75,616/6,890 (9.1)	NR	NR
	Blankenburg, et al. [32]	Patients with CKD and T2D	Within 60 days	Spironolactone	5,465/1,634 (29.9)	NR	Spironolactone non-users	5,465/1,503 (27.5)	NR	NR
				Non-persistent users	2,630/800 (30.4)	NR				
				Persistent users	2,800/680 (24.3)	NR				
Cumulative incidence of hyperkalemia**	Yamazaki, Yoshihara [25]	Patients with CKD in diabetes	NR	MRAs	2295/175 (7.63)	NR	NR	NR	NR	NR

*CI* confidence interval, *CKD* chronic kidney disease, *FU* follow-up, *HF* heart failure, *HR* hazard ratio, *MI* myocardial infarction, *MRAs* mineralocorticoid receptor antagonists, *N*/*n* number of patients/number of patients with an event, *NR* not reported, *PY* person-years, *T2D* type 2 diabetes

*Length of follow-up not reported, however, the observation period was 5 years

**Definition not reported

^p for interaction (history of diabetes mellitus—Yes vs No)

### RWE in subgroups of CKD and HF (with or without diabetes)

The effectiveness of MRAs was reported in 11 studies (11 publications, Table 1) in patients with CKD and HF, while safety was reported in four studies. One study reported results for both patients with CKD and HF, as well as in CKD and T2D and HF [14]. Most studies considered both HF with preserved and reduced ejection fraction, only 2 studies reported results of HF with reduced ejection fraction [24, 34]. Of note, Oh et al. reported that no difference in clinical outcomes and significance in interaction with spironolactone use could be identified between both HF types [21]. Again, the definition of the CKD population varied among the included studies (Supplementary Table 1).

### Effectiveness

#### Renal outcomes

Renal outcomes were retrieved only from the study by Mavrakanas et al. [12] There was a similar incidence of eGFR decline of > 30% from baseline among patients treated with MRAs compared to a non-MRA population; however, the difference was not statistically significant (22.6% vs. 19.7%, respectively, HR = 1.2, 95% CI [0.77–1.88] not adjusted; HR = 1.1, 95% CI [0.62–1.98] after adjustment for NYHA stage ≥ III, systolic blood pressure, baseline left ventricular ejection fraction, diuretic use, and the propensity score for MRA use) [12]. There was also a lower incidence of doubling of serum creatinine among patients treated with MRAs compared to MRA non-users, however, it was not statistically significant [12]. The risk of a composite outcome of serious hyperkalemia or any doubling of serum creatinine in patients not yet on dialysis was similar in both MRA users and non-users [12].

#### CV outcomes

In the study by Inampudi et al., the incidence of hospitalization for HF was similar between spironolactone users and non-users, and the incidence was 44% vs. 40% in patients with HF and advanced CKD [24]. In patients with pre-dialysis CKD stage 5 and both mild and severe HF, the hazard of hospitalization for HF was higher but not significantly, among spironolactone users compared to non-users.

Blankenburg et al. reported that the incidence of myocardial infarction was numerically higher among patients treated with spironolactone (more and less than six months) compared to the non-MRA treatment arm, whereas a similar percentage of patients in both treatment arms experienced strokes. However, no statistical tests were performed [14].

#### All-cause mortality

In the Löfman et al. study [35], patients with CKD receiving MRAs were at a statistically significant lower risk of all-cause mortality compared to MRA non-users (event rates during three years of follow up: 21.5 vs. 26.9/100 person-years, respectively, p < 0.001), whereas in the Tseng et al. study [19], spironolactone use was related to a significantly higher risk of all-cause mortality compared to spironolactone non-users, irrespective of the severity of HF. In two other studies (Martinez-Milla et al. [36], Devesa et al. [34]), a numerically greater risk of all-cause mortality associated with MRA use compared to non-MRA use was shown, however, the results presented in these studies were not statistically significant. In the Inampudi et al. study [24], there was no statistical difference between the incidence of all-cause death between spironolactone users and non-users (49% vs. 46%, respectively, p = 0.441).

#### All-cause hospitalization

Two studies reported all-cause hospitalization defined as rehospitalization. Both included patients with HF and CKD [21, 24]. In one study, spironolactone use was associated with a statistically significant higher incidence of all-cause hospitalization compared to spironolactone non-users (77% vs 70%, p = 0.001 post score matching [24]). Subgroups of patients with eGFR < 15 mL/min/1.73 m^2^ and eGFR 15–45 mL/min/1.73 m^2^ receiving spironolactone after one year post-discharge were at higher risk of all-cause readmission in comparison to spironolactone non-users [24].

The results for effectiveness in a subgroup of CKD and HF are shown in Table 5.

**Table 5:** Effectiveness outcomes: CKD and HF

Outcome		Author and name	Population	Timepoint	MRAs			Controls			HR [95% CI], p-value
					Treatment arm	N/n (%)	Event rate/ 1000 PY	Reference	N/n (%)	Event rate/1000 PY	
*Composite*											
Death from any cause, MI, or admission for decompensated HF		Mavrakanas, Giannetti [12]	Patients with HF and CKD	NR	MRAs	119/48 (40.3)	NR	Non-MRA users	397/154 (38.8)	NR	1.13 [0.82; 1.57]^NA^ 1.04 [0.69; 1.56]^A^
Serious hyperkalemia (K+ > 6 mmol/L) or any doubling of serum creatinine in patients not yet on dialysis						115/8 (7)	NR		358/25 (7)	NR	1.06 [0.48; 2.34]^NA^ 0.82 [0.25; 2.66]^A^
Death of any cause or admission due to HF		Martinez-Milla, Garcia [36]	Patients ≥ 75 years with LVEF ≤ 35%* and with CKD	NR	MRAs	NR	NR	Non-MRA users	NR	NR	1.08 [0.84; 1.38]^U^
Major CV events (death or HHF)		Devesa, Cortes Garcia [34]	Patients aged ≥ 75 years with LVEF ≤ 35% (HFrEF)	NR	MRAs	NR	NR	Non-MRA users	NR	NR	1.08 [0.84; 1.39]▲
All-cause mortality or readmission		Inampudi, Parvataneni [24]	Patients with HFrEF (EF < 45%) and advanced CKD	1 year post-discharge	Spironolactone	207/189 (91)	NR	Non-Spironolactone users	933/779 (84)	NR	1.22 [1.04; 1.42]^NA^, p = 0.014 1.3 [1.09; 1.54]^P^, p = 0.003
*Renal*											
eGFR drop 30%		Mavrakanas, Giannetti [12]	Patients with HF and CKD	NR	MRAs	115/26 (22.6)	NR	Non-MRA users	361/71 (19.7)	NR	1.2 [0.77; 1.88]^NA^ 1.1 (0.62; 1.98]^A^
Persistent creatinine doubling		Mavrakanas, Giannetti [12]	Patients with HF and CKD	NR	MRAs	115/2 (1.7)	NR	Non-MRA users	358/17 (4.7)	NR	0.4 [0.09; 1.71]^NA^ 0.33 [0.04; 2.78]^A^
*Cardiovascular*											
HHF ■		Tseng, Liu [19]	Patients with CKD in pre-dialysis stage 5 with mild prior HF	NR	Spironolactone	2880/NR	NR	Non-Spironolactone users	NR	NR	1.35 [0.9; 2.01]^A^
			Patients with CKD in pre-dialysis stage 5 with severe prior HF	NR	Spironolactone	870/NR	NR	Non-Spironolactone users	NR	NR	1.31 [0.8; 2.14]^A^
HHF		Inampudi, Parvataneni [24] **	Patients with HFrEF (EF < 45%) and advanced CKD	1 year post-discharge	Spironolactone	207/91 (44)	NR	Non-Spironolactone users	933/370 (40)	NR	1.14 [0.91; 1.44]^NA^, p = 0.16 1.02 [0.8; 1.3]^P^, p = 0.847
HF		Blankenburg, Fett [14]	Patients with CKD and HF	< 6 months	Spironolactone	512/470 (91.8)	NR	Non-MRA users	14,653/11,274 (76.9)	NR	NR
				≥ 6 months	Spironolactone	373/351 (94.1)	NR	Non-MRA users	14,653/11,274 (76.9)	NR	NR
			Patients with CKD and T2D and HF	< 6 months	Spironolactone	808/765 (94.7)	NR	Non-MRA users	21,144/16,511 (78.1)	NR	NR
				≥ 6 months	Spironolactone	458/425 (92.8)	NR	Non-MRA users	21,144/16,511 (78.1)	NR	NR
MI		Blankenburg, Fett [14]	Patients with CKD and HF	< 6 months	Spironolactone	512/118 (23)	NR	Non-MRA users	14,653/2185 (14.9)	NR	NR
				≥ 6 months	Spironolactone	373/90 (24.1)	NR	Non-MRA users	14,653/2185 (14.9)	NR	NR
			Patients with CKD and T2D and HF	< 6 months	Spironolactone	808/218 (27.0)	NR	Non-MRA users	21,144/4098 (19.4)	NR	NR
				≥ 6 months	Spironolactone	458/124 (27.1)	NR	Non-MRA users	21,144/4098 (19.4)	NR	NR
Stroke		Blankenburg, Fett [14]	Patients with CKD and HF	< 6 months	Spironolactone	512/125 (24.4)	NR	Non-MRA users	14,653/3249 (22.2)	NR	NR
				≥ 6 months	Spironolactone	373/78 (20.9)	NR	Non-MRA users	14,653/3249 (22.2)	NR	NR
			Patients with CKD and T2D and HF	< 6 months	Spironolactone	808/219 (27.1)	NR	Non-MRA users	21,144/5307 (25.1)	NR	NR
				≥ 6 months	Spironolactone	458/99 (21.6)	NR	Non-MRA users	21,144/5307 (25.1)	NR	NR
*All-cause mortality*											
All-cause mortality		Martinez-Milla, Garcia [36]	Patients ≥ 75 years with LVEF ≤ 35%* and with CKD	NR	MRAs	NR	NR	Non-MRA users	NR	NR	1.09 [0.83; 1.44]^U^
		Lofman, Szummer [35]	Patients with MI and HF registered in the Swedish MI registry, CKD subgroup	Up to 3 years	MRAs	1802/NR	21.5 (20.0; 23.1)^	Non-MRA users	1,386/NR	26.9 (26.4; 27.5)^	0.92 [0.85; 0.99]^A^, p < 0.001***
Association of MRA treatment with total mortality		Devesa, Cortes Garcia [34]	Patients aged ≥ 75 years that had an LVEF ≤ 35% (HFrEF), CKD subgroup	NR	MRAs	NR	NR	Non-MRA users	NR	NR	1.09 [0.83; 1.44]▲
All cause-mortality		Tseng, Liu [19]	Patients with CKD in pre-dialysis stage 5 with mild prior HF	NR	Spironolactone	2880/NR	NR	Non-Spironolactone users	NR	NR	1.39 [1.16; 1.67]^A^
			Patients with CKD in pre-dialysis stage 5 with severe prior HF	NR	Spironolactone	870/NR	NR	Non-Spironolactone users	NR	NR	1.38 [1.04; 1.82]^A^
All-cause mortality (KM)		Oh, Kang [21]	Patients with ADHF and severe renal dysfunction	NR	Spironolactone	NR/NR (18.7)	NR	Non-Spironolactone users	NR/NR (25.3)	NR	0.698 [0.525; 0.928]^NA^, p = 0.013 (log rank) 0.974 [0.681; 1.392]^A^, p = 0.884
All-cause mortality■				NR	Spironolactone	105/NR (19)	NR	Non-Spironolactone users	105/NR (25.7)	NR	0.631 [0.354; 1.125]^NA^, p = 0.013 (log rank) 0.646 [0.352; 1.185]^A^, p = 0.158
All-cause mortality		Inampudi, Parvataneni [24]	Patients with HFrEF (EF < 45%) and advanced CKD	1 year post-discharge	Spironolactone	207/102 (49)	NR	Non-Spironolactone users	933/432 (46)	NR	1.09 [0.88; 1.35]^NA^, p = 0.441 1.05 [0.83; 1.31]^P^, p = 0.706
All-cause mortality		Lin, Yu [53]	Patients with HF and CKD	NR	Spironolactone	430/NR	NR	Non-Spironolactone users	NR	NR	0.522 [0.366; 0.744], p < 0.001
All-cause hospitalization											
Re-hospitalization ■		Oh, Kang [21]	Patients with ADHF and severe renal dysfunction	NR	Spironolactone	105/42 (39.5)	NR	Non-Spironolactone users	105/37 (35.7)	NR	p = 0.724 (log rank)
All-cause readmission		Inampudi, Parvataneni [24]	Patients with HFrEF (EF < 45%) and advanced CKD	1-year post-discharge	Spironolactone	207/160 (77)	NR	Non-Spironolactone users	933/656 (70)	NR	1.24 [1.04; 1.48]^NA^, p = 0.014 1.36 [1.13; 1.63]^P^, p = 0.001

*A* adjusted, *ADHF* acute decompensated heart failure, *CI* confidence interval, *CKD* chronic kidney disease, *CV* cardiovascular, *eGFR* estimated glomerular rate, *HF* heart failure, *HFrEF* heart failure with reduced ejection fraction, *HHF* hospitalization for heart failure, *HR* hazard ratio, *KM* Kaplan Meier survival curves, *LVEF* left ventricular ejection fraction, *MI* myocardial infarction, *MRAs* mineralocorticoid receptor antagonists, *N/n* number of patients/number of patients with event, *NA* not adjusted, *NR* not reported, *P* after propensity score matching, *PY* person-years, *U* univariate analysis

*As measured by a 2-dimensional echocardiogram

**HF readmission

***Adjusted mortality for MRA use

^Per 100 person-years

▲Odds ratio

■Results in the matched cohort

### Safety

None of the included studies compared the rates of hyperkalemia between MRA users and non-users. The overall incidence of hyperkalemia among patients treated with MRAs with CKD and HF ranged from 7.5% [37] to 15.2%. [14]; however, Buckallew et al. [38] showed its relationship with EF, with patients with EF > 40% having higher rates of hyperkalemia than those with EF ≤ 40% (9.52% vs 3.44%). [38] In the Blankenburg et al. study[14], the incidence of hyperkalemia was driven by the presence of HF and T2D and was higher in MRA users than in non-users, especially in patients with a short duration of spironolactone use [14]. In the Trevisan et al. study [22], patients with CKD and HF receiving MRAs were at statistically significantly higher risk of any, mild or moderate/severe hyperkalemia compared to patients without CKD at baseline, irrespective of the baseline eGFR level [22].

The results for safety in a subgroup of CKD and HF are shown in Table 6.

**Table 6:** Results on hyperkalemia in CKD and HF

Author and name	Outcome definition	Population	Timepoint	MRAs			Controls			HR [95% CI], p-value
				Treatment arm	N/n (%)	Event rate/1000 PY	Reference	N/n (%)	Event rate/1000 PY	
Hyperkalemia										
Buckallew et al. [38]	Serum potassium ≥ 5.5 mEq/L	Patients with CKD and HF with EF > 40%	During FU of 12–18 months	Spironolactone	63/6 (9.52)	NR	-	-	-	-
		Patients with CKD and HF with EF ≤ 40%			58/2 (3.44)					
Blankenburg, Fett [14]	NR	Patients with CKD and HF	< 6 months	Spironolactone	512/78 (15.2)	NR	Non-MRA users	14,653/1593 (10.9)	NR	NR
			≥ 6 months		373/53 (14.2)	NR		14,653/1593 (10.9)	NR	NR
		Patients with CKD and T2D and HF	< 6 months	Spironolactone	808/190 (23.5)	NR	Non-MRA users	21,144/3647 (17.2)	NR	NR
			≥ 6 months		458/75 (16.4)	NR		21,144/3647 (17.2)	NR	NR
Trevisan, de Deco [22]	Predictors of any hyperkalemia risk ( K+ > 5.0 mmol/L)	Patients with CKD and eGFR 45–60 mL/min/1.73 m^2^	At baseline (FU: 1 year)	MRA users (spironolactone, eplerenone)	NR	NR	No CKD (eGFR > 60 mL/min/1.73m^2^)	NR	NR	1.39 [1.23; 1.57]
		Patients with CKD and eGFR 30–45 mL/min/1.73 m^2^								1.95 [1.7; 2.24]
		Patients with CKD and eGFR < 30 mL/min/1.73 m^2^								2.43 [1.98; 2.98]
	Predictors of mild hyperkalemia risk ( K+ > 5.0–5.5 mmol/L)	Patients with CKD and eGFR 45–60 mL/min/1.73 m^2^	At baseline (FU: 1 year)	MRA users (spironolactone, eplerenone)	NR	NR	No CKD (eGFR > 60 mL/min/1.73m^2^)	NR	NR	1.32 [1.15; 1.51]
		Patients with CKD and eGFR 30–45 mL/min/1.73 m^2^								1.7 [1.46; 1.98]
		Patients with CKD and eGFR < 30 mL/min/1.73 m^2^								1.85 [1.45; 2.35]
	Predictors of moderate/severe hyperkalemia risk ( K+ > 5.5 mmol/L.)	Patients with CKD and eGFR 45–60 mL/min/1.73 m^2^	At baseline (FU: 1 year)	MRA users (spironolactone, eplerenone)	NR	NR	No CKD (eGFR > 60 mL/min/1.73m^2^)	NR	NR	1.63 [1.34; 1.97]
		Patients with CKD and eGFR 30–45 mL/min/1.73 m^2^								2.4 [1.94; 2.96]
		Patients with CKD and eGFR < 30 mL/min/1.73 m^2^								3.68 [2.76; 4.9]
	Predictors of any hyperkalemia risk ( K+ > 5.0 mmol/L)*	Patients with CKD and eGFR 45–60 mL/min/1.73 m^2^	At baseline (FU: 1 year)	MRA users (spironolactone, eplerenone)	NR	NR	No CKD (eGFR > 60 mL/min/1.73m^2^)	NR	NR	1.41 [1.23; 1.61]
		Patients with CKD and eGFR 30–45 mL/min/1.73 m^2^								2.12 [1.83; 2.45]
		Patients with CKD and eGFR < 30 mL/min/1.73 m^2^								2.69 [2.17; 3.33]
	Predictors of mild hyperkalemia risk ( K+ > 5.0–5.5 mmol/L)*	Patients with CKD and eGFR 45–60 mL/min/1.73 m^2^	At baseline (FU: 1 year)	MRA users (spironolactone, eplerenone)	NR	NR	No CKD (eGFR > 60 mL/min/1.73m^2^)	NR	NR	1.3 [1.12; 1.51]
		Patients with CKD and eGFR 30–45 mL/min/1.73 m^2^								1.82 [1.54; 2.15]
		Patients with CKD and eGFR < 30 mL/min/1.73 m^2^								1.96 [1.51; 2.54]
	Predictors of moderate/severe hyperkalemia risk ( K+ > 5.5 mmol/L.)*	Patients with CKD and eGFR 45–60 mL/min/1.73 m^2^	At baseline (FU: 1 year)	MRA users (spironolactone, eplerenone)	NR	NR	No CKD (eGFR > 60 mL/min/1.73m^2^)	NR	NR	1.69 [1.37; 2.08]
		Patients with CKD and eGFR 30–45 mL/min/1.73 m^2^								2.67 [2.13; 3.36]
		Patients with CKD and eGFR < 30 mL/min/1.73 m^2^								4.23 [3.13; 5.7]
Cooper, Hammill [37]	Serum potassium > 5.5 mEq/L	High risk patients with HF and CKD	Days 11 through 90 after MRA initiation	MRAs	440/33 (7.5)	NR	NR	NR	NR	NR
Oh, Kang [21]	Serum potassium ≥ 5.5 mmol/L	Patients with ADHF and severe renal dysfunction	At 1 month after discharge	Spironolactone	NR/NR (10.3)	NR	NR	NR	NR	NR

*ADHF* acute decompensated heart failure, *CI* confidence interval; *CKD* chronic kidney disease, *HF* heart failure, *HR* hazard ratio, *MRAs* mineralocorticoid receptor antagonists, *NR* not reported

*On-therapy design

### RWE in subgroups of CKD and a history of CVD

Only one study conducted by Tseng et al. [19] among patients with CKD in pre-dialysis stage 5 reported results in subgroups of patients stratified by CVD events, including the presence or absence of coronary artery disease, atrial fibrillation and stroke [19]. Results for those subgroups were available with regard to hospitalization for HF and to all-cause mortality (Table 7).

**Table 7:** Effectiveness outcomes: CKD and CVD history

Outcome	Author and name	Population	Timepoint	MRAs			Controls			HR [95% CI], p-value
				Treatment arm	N/n (%)	Event rate/1000 PY	Reference	N/n (%)	Event rate/1000 PY	
*Cardiovascular*										
HHF■	Tseng, Liu [19]	Patients with CKD in pre-dialysis stage 5 with CAD	NR	Spironolactone	6983/NR	NR	Non-Spironolactone users	NR	NR	1.37 [1.03; 1.83]^A^
		Patients with CKD in pre-dialysis stage 5 with stroke	NR	Spironolactone	4996/NR	NR	Non-Spironolactone users	NR	NR	1.45 [0.94; 2.26]^A^
		Patients with CKD in pre-dialysis stage 5 with AF	NR	Spironolactone	566/NR	NR	Non-Spironolactone users	NR	NR	1.75 [0.77; 3.97]^A^
*All-cause mortality*										
All cause-mortality■	Tseng, Liu [19]	Patients with CKD in pre-dialysis stage 5 with CAD	NR	Spironolactone	6983/NR	NR	Non-Spironolactone users	NR	NR	1.32 [1.16; 1.51]^A^
		Patients with CKD in pre-dialysis stage 5 with stroke	NR	Spironolactone	4996/NR	NR	Non-Spironolactone users	NR	NR	1.27 [1.08; 1.48]^A^
		Patients with CKD in pre-dialysis stage 5 with AF	NR	Spironolactone	566/NR	NR	Non-Spironolactone users	NR	NR	1.66 [1.14; 2.41]^A^

*A* adjusted, *AF* atrial fibrillation, *CAD* coronary artery disease, *CI* confidence interval, *CKD* chronic kidney disease, *CVD* cardiovascular disease, *HR* hazard ratio, *N/n* number of patients/number of patients with event, *NR* not reported

■ Results in the matched cohort

#### Hospitalization for HF

The risk of hospitalization for HF was significantly higher in spironolactone users compared to non-users only in the subgroup of patients with a history of coronary artery disease (HR = 1.37, 95% CI [1.03; 1.83]) [19].

#### All-cause mortality

The use of spironolactone was associated with a significantly higher risk of all-cause mortality when compared with non-use of spironolactone in each subgroup of CV events [19].

## Discussion

An important finding from this review is the low availability of RWE for MRAs, especially among patients with CKD and diabetes, even though diabetes is the most significant contribution to the global CKD burden. Limited robust data on the efficacy and safety of steroidal MRAs in CKD is on one hand due to the focus on CV outcomes, but on the other hand, due to the fact that most existing studies were not powered to detect hard primary outcomes including mortality or long-term renal outcomes. Moreover, among the included studies, only around half of the cohort studies were of good quality, while the remaining studies were rated as fair or poor quality. On top of this, a notable lack of studies with robust population sizes was noted. In the overall CKD population, MRAs appeared to have a limited effect on renal outcomes; a significant or sustained eGFR reduction was observed but available data suggest no efficacy in delaying progression to ESKD. No CV protection, determined as a reduction in major adverse cardiovascular event rates and the occurrence of CV death, was revealed by RWE studies. Results for all-cause mortality and hospitalization for HF were inconsistent as well; however, evidence from studies with the longest follow-up indicate similar or lower incidence for spironolactone non-users. In terms of safety, most results consistently reported a higher incidence of hyperkalemia among patients on MRAs in all CKD stages, regardless of the hyperkalemia definitions considered by the authors. The association of MRAs with a range of side effects and adverse events translated into high rates of discontinuation reaching 44% [14] in the real-world setting in contrast to clinical trials, where observed rates can be as low as 2% [39, 40]. For the subgroup of patients with CKD and diabetes (especially T2D), the evidence was low and limited to retrospective studies of poor quality. The covered outcomes were sparse and limited to all-cause mortality, persistence and discontinuation of treatment, hyperkalemia, and CV outcomes. However, while limited data exist regarding CKD patients, adverse events are seen to play a significant role in the discontinuation of treatment with MRAs.

The Cochrane review, conducted by Chung et al. [15], concluded that there is consistent evidence from RCTs supporting the use of MRAs in patients with CKD, as MRAs may reduce proteinuria, eGFR, and systolic blood pressure in adults who have mild-to-moderate CKD. At the same time, MRAs may increase the risk of hyperkalemia, acute kidney injury and gynecomastia when added to an ACEi and/or ARB, and the effects of MRAs added to an ACEi and/or ARB on the risks of death, major cardiovascular events, and kidney failure in people with proteinuric CKD are uncertain. However, long-term data on the effectiveness and safety of MRAs, especially on major patient-centered outcomes such as death, kidney failure or CV events were insufficiently reported in RCTs. Moreover, results from RCTs may differ from those observed in the real world. A surprising finding from a review of real-world studies conducted in this paper was the association of MRAs with a higher incidence of myocardial infarction, stroke and HF while data from RCTs indicated only uncertain effects on CV events. The incidence of hyperkalemia was shown to be higher in MRA users than in the overall CKD population. Chung et al. [15] found that MRAs were also associated with acute kidney injury and gynecomastia, however, such data were not available in the studies included in our review.

More evidence is available for the CKD population with HF. Khan et al. [41] conducted a systematic literature review evaluating the efficacy and safety of MRAs in patients with CKD and HF with only three observational studies meeting inclusion criteria. The authors did not identify benefits related to treatment with MRAs, however, the conclusions from these studies were limited due to residual confounding and concern for bias [41]. Our review of 21 literature reports showed that in this subgroup, treatment with MRAs had an unclear effect on hospitalization for HF. We also found inconsistent results for all-cause mortality, with most studies reporting no significant difference in the incidence of all-cause death between MRA users and non-users. The incidence of hyperkalemia was found to be driven by the presence of HF and T2D, and was higher in MRA users than in non-users, especially in patients with a short duration of spironolactone use [14, 33].

Our review and other reports from the literature suggest that treatment with MRAs is associated with unclear renal benefits and insufficient CV protection; however, they considered only steroidal MRAs. Nonsteroidal selective MRAs have been shown to delay chronic kidney disease progression and to reduce the occurrence of CV events in patients with CKD and T2D when compared with placebo [42–44]. Recent head-to-head comparisons of pharmacological and clinical characteristics between steroidal and non-steroidal MRAs concluded that both agents exhibit comparable efficacy but the safety of nonsteroidal MRAs may be superior in high-risk patients suffering from CKD and/or HF [45]. Additional cardiorenal benefits of nonsteroidal MRAs may be rooted in their natriuretic effect as well as anti-inflammatory and antifibrotic actions resulting from selective inhibition of overactivation of the mineralocorticoid receptor which may further translate into tissue remodeling [43]. These antifibrotic and anti-inflammatory properties of nonsteroidal MRAs were not seen for classical steroidal MRAs, but results were observed in the experimental animal model. [46].

Gaps identified in this review showed the lack of sufficient evidence that particularly applied to renal outcomes. No effectiveness and safety data, such as a change in eGFR, urinary albumin-to-creatinine ratio, kidney failure, injury and death, the occurrence of fatal adverse events and adverse events leading to discontinuation of treatment, as well as health-related quality of life in the population of patients with CKD treated with MRAs were available. However, there are several ongoing and planned analyses on steroidal MRAs in patients with CKD and T2D, which may address the outlined efficacy and safety gaps in this population. The BARACK D trial is an ongoing randomized, open, blinded end-point, 36-month study that aims to evaluate the efficacy of spironolactone in a patient with stage 3b CKD and place the focus on cardio-protection and a delay of renal impairment [47]. The DISCOVER CKD global observational study aims to investigate the clinical and financial burden of CKD in the real-world setting by focusing on treatment patterns, effectiveness, patient outcomes and patient quality of life [9]. The Health ABC Study investigated the degree of kidney disease progression in community-living elders mainly focusing on safety [48]. Acquiring additional evidence will support a choice of treatment in patients with CKD and T2D.

This review is a comprehensive review of effectiveness and safety real-world data for MRAs among a broad population of patients with CKD. This approach aimed to collect all the relevant studies (e.g., studies conducted among patients with CKD and T2D, but also conducted in overall CKD and reporting data for subgroups of patients with T2D). It was conducted based on a predefined protocol with clear inclusion and exclusion criteria and followed the Cochrane guidelines for systematic review reporting. A comprehensive search strategy was used to reduce reporting bias in the review process. To minimize publication bias, we did not apply any restrictions in terms of language and any specific geographical scope.

Although this review was conducted with strict selection criteria, there were several potential limitations. Even though the search strategy was not limited to steroidal MRAs, no real-world study for non-steroidal MRAs was identified. One of the main limitations was the large heterogeneity of studies. First, this heterogeneity was evident in terms of CKD definition. Studies with a population described simply as CKD, DKD or diabetic nephropathy were included in this systematic literature review, meaning it may have a component of publication bias. The applied CKD criteria differed among studies while, in some studies, the CKD definition was not provided at all. Additionally, heterogeneity was present in terms of the patients’ baseline characteristics. That is, baseline characteristics, namely in CKD severity differed considerably among studies. For example, Blankenburg et al. [14] enrolled patients irrespective of CKD severity, resulting in about 20% of patients with ESKD being included. However, CKD stage 3 was the most common stage identified at baseline in other identified studies.

The main limitation of most studies included in this review was their retrospective design or reliance on historical databases. Residual confounding occurring in such studies could lead to an over- or underestimation of the observed association between the use of MRAs and assessed patient-centered outcomes. In addition, the robustness of the results may be limited by the lack of availability of data, medical records entry errors, or coding limitations. Moreover, around half of the studies were assessed as having fair or poor quality, driven mainly by the poor comparability between cohorts or by insufficient information on all domains to make an assessment. Furthermore, due to the considerable heterogeneity among the included studies regarding the population (CKD definitions, baseline characteristics, namely in CKD severity), as well as the follow-up and outcome definitions, conducting a meta-analysis was not feasible.

## Conclusions

Although our comprehensive review of real-world data revealed limited availability of evidence on the effectiveness and safety of MRAs in patients with CKD, as well as in subgroups with diabetes, HF or history of CVD, they were shown to have a limited effect on renal and CV outcomes in these patients. Moreover, identified studies were small and non-rigorous, resulting in a notable lack of evidence in this population. Gaps in the evidence regarding the efficacy and safety of MRAs are particularly relevant in patients with CKD and diabetes, therefore, further research is warranted.

## Supplementary Information

Below is the link to the electronic supplementary material.

Supplementary file1 (DOCX 111 KB)
