# Supplementary material for: Real-world evidence for steroidal mineralocorticoid receptor antagonists in patients with chronic kidney disease
Source: J Nephrol. 2022 Nov 23;36(4):1135–67. doi: 10.1007/s40620-022-01492-w (PMC10227157; doi:10.1007/s40620-022-01492-w)
Supplement: Supplementary file 1 — Supplementary file1 (DOCX 111 KB) [file 40620_2022_1492_MOESM1_ESM.docx]

**Supplementary Table 1: Population definition and characteristics of included studies**

| **Author, year** | **Country** | **Type of study** | **Interventions** | **Sample size*** | **Follow-up** | **Population** | **Definition of CKD** |
| --- | --- | --- | --- | --- | --- | --- | --- |
| **Buckallew, et al. [**[**38**](#_ENREF_38)**]** | USA | Single centre, retrospective cohort study | Spironolactone | 121 (121) | 12-18 months | Patients with HF and CKD stages 3-5 | According to the Kidney Disease: Improving Global Outcomes (KDIGO):  stage 3 eGFR 30-59 mL/min/1.73 m^2^  stage 4 eGFR 15-29 mL/min/1.73 m^2^  stage 5 eGFR <15mL/min/1.73 m^2^ or on hemodialysis or peritoneal dialysis |
| **Blankenburg, et al. [**[**30**](#_ENREF_30)**]** | Germany | Single centre, retrospective cohort study | •MRAs  •No MRAs | 11,554 (5,777) | 1 year | Adult CKD patients, MRA users and MRA non-users | Based on the presence of ICD 10GM codes |
| **Blankenburg, et al. [**[**32**](#_ENREF_32)**]** | USA | Multicentre, retrospective cohort study | •Spironolactone  •No MRA | 10,930 (5,465) | 1 year | Patients with CKD and T2D | NR |
| **Gay, et al. [**[**49**](#_ENREF_49)**]** | USA | Multicentre, retrospective cohort study | •MRAs  •No MRAs | 235,032 (5,889) | NR | Patients aged ≥18 years with a first diagnosis of CKD and a first prescription for steroidal MRAs | NR |
| **Giannetti, et al. [**[**50**](#_ENREF_50)**]** | NR | Retrospective cohort study | MRA | 1430 (314) | NR | Patients with HF with or without CKD | NR |
| **Qu, et al. [**[**20**](#_ENREF_20)**]** | China | Single centre, retrospective registry-based cohort study | •Spironolactone  •Standard treatment | 560 (200) | 30 months | Patients with AMI and CKD | eGFR <60 mL/min/1.73 m^2^ |
| [**Mavrakanas, Giannetti [12]**](#_ENREF_12) | NR | Single centre, retrospective cohort study | •Spironolactone  •Eplerenone  •No MRA | 1,430 (314) | NR | Patients with HF with and without CKD | eGFR <60 mL/min/1.73 m^2^ |
| [**Blankenburg, Fett [14]**](#_ENREF_14) | USA | Multicentre, retrospective cohort study | •Spironolactone**  •No MRA | 229,004 (5899) | 5 years | •Patients aged ≥18 years with a first diagnosis of CKD •Patients with a first prescription for steroidal MRAs | CKD population comprised of patients who had received a diagnosis of CKD after the start of the observation period in a time window that allowed for at least one year of data coverage before the diagnosis date (inclusion date) and for at least one year of data coverage after this date. This included patients diagnosed for the first time and those who may have received a repeat diagnosis. |
| [**Jun, Jardine [31]**](#_ENREF_31) | Australia | Multicentre, retrospective population-based cohort study | •Spironolactone  •Eplerenone | 20,184 (1648) | 3.9 (2.2; 4.3)† years | Adult patients with CKD who were prescribed a RAAS | eGFR <60 mL/min/1.73 m^2^ at index date |
| [**Martinez-Milla, Garcia [36]**](#_ENREF_36) | Spain | Single centre, retrospective cohort study | •MRAs  •ACEIs/ARBs | 390 (156) | 32 (23)‡ months | Patients 75 years of age or older with LVEF ≤35% and with CKD | Renal impairment: GFR <60 mL/min/1.73 m^2^ |
| [**Yamazaki, Yoshihara [25]**](#_ENREF_25) | Japan | Retrospective cohort study | MRAs | 19,582 (2295) | Observation period max. of 8 years | Patients with CKD and diabetes | eGFR <60 mL/min/1.73m2 at index date |
| [**Blankenburg, Kovesdy [33]**](#_ENREF_33) | USA | Multicentre, retrospective cohort study | •Spironolactone  •Eplerenone | 10,930 (5465) | 1 year | Patients with CKD and T2D | NR |
| [**Linde, McEwan [52]**](#_ENREF_52) | UK | Retrospective observational, database-analysis | •MRAs  •ACEIs  •ARBs | 100,572 (9687) | NR | Patients with CKD stage 3+ | eGFR 60 mL/min/1.73m^2^ or a diagnosis of CKD stage 3+ |
| [**Lofman, Szummer [35]**](#_ENREF_35) | Sweden | Multicentre, prospective registry | •Spironolactone  •Eplerenone | 45,071 (4470) | Up to 3 years⁺ | Patients with MI and HF registered in the Swedish MI registry, with CKD■ | eGFR <60 mL/min/1.73 m^2^ |
| [**Trevisan, de Deco [22]**](#_ENREF_22) | Sweden | Multicentre, prospective cohort study | •Spironolactone  •Eplerenone  •Beta-blocker users | 13,726 (3788)^ | 1 year | All residents in the region of Stockholm, undertaking at least one measurement of plasma creatinine in ambulatory or hospital care during 2006–2011, with CKD■ | eGFR <60 mL/min/1.73 m^2^ |
| [**Yang, Kor [26]**](#_ENREF_26) | Taiwan | Retrospective population-based cohort study | •Spironolactone  •Spironolactone non-users | 14,669 (785) | •Spironolactone users: 3.57 (3.2)‡ years •Spironolactone non-users: 3.24 (3.23)‡ years | Patients with CKD stage 3-4 | •Patients with CKD were defined as having at least one record of a CKD diagnostic code made by a nephrologist per month for at least three consecutive months.  •Incident CKD patients represented moderate to severe (stage 3–4) CKD patients. |
| [**Cooper, Hammill [37]**](#_ENREF_37) | USA | Multicentre, retrospective cohort study | •Eplerenone  •Spironolactone | 10,443 (446)^ | •The early FU testing period; 10 days after the index date  •The extended FU testing period; days 11 through 90 after MRA initiation | High risk patients with HF■ | eGFR <60 mL/min/1.73 m^2^ |
| [**Devesa, Cortes Garcia [34]**](#_ENREF_34) | Spain | Prospective observational study | •MRAs  •No MRAs | 802 (390)^ | 32 (23)‡ months | Patients aged ≥75 years that had an LVEF ≤35% (HFrEF)■ | NR |
| [**Gillis, Lees [28]**](#_ENREF_28) | UK | Retrospective cohort study | •Spironolactone  •Eplerenone  •No MRAs | 7,766 (402) | 2,269† days | Patients with CKD treated or not treated with MRA | NR |
| [**Tseng, Liu [19]**](#_ENREF_19) | Taiwan | Multicentre, retrospective population-based longitudinal cohort study | •Spironolactone  •Standard of care | 27,213 (1363) | •85,758 PY  •31 (14; 57)† months | Patients with CKD in pre-dialysis stage 5 | Serum creatinine levels ≥530.4 μmol/L (approximately eGFR <15 mL/min/1.73 m^2^) |
| [**Oh, Kang [21]**](#_ENREF_21) | Korea | Multicentre, retrospective cohort study | •Spironolactone  •No spironolactone | 1035 (105) | 407 (108; 905)† days | Patients with ADHF and severe renal dysfunction■ | eGFR <45 mL/min/1.73 m^2^ |
| [**Hassan, Qureshi [27]**](#_ENREF_27) | USA | Retrospective case-control study | •Spironolactone  •Control | 850 (425) | 5 years | Adult (≥18 years) ambulatory patients who were diagnosed with CKD stage 2 or worse as defined by the CKD-EPI equation | CKD stage 2 or worse as defined by the CKD-EPI equation |
| [**Inampudi, Parvataneni [24]**](#_ENREF_24) | USA | Multicentre, cohort study | •Spironolactone  •No spironolactone | 1,140 | 1 year | Patients with HFrEF (EF <45%) and advanced CKD | Advanced CKD defined as eGFR <45 mL/min/1.73 m^2^ |
| [**Lin, Yu [53]**](#_ENREF_53) | Taiwan | Single centre, prospective cohort study | MRAs | 801 (430)^ | 3 years | Patients with HF (LVEF ≤ 35%) and CKD■ | NR |
| [**Herget-Rosenthal, Dehnen [29]**](#_ENREF_29) | Germany | Multicentre, retrospective cohort study | Aldosterone antagonists*** | 803 (204)^ | 3 years | Patients with or at high risk of CKD exclusively managed in primary care■ | CKD or high risk of CKD were defined as:  •eGFR <60 mL/min/1.73 m^2^  •Arterial hypertension for ≥5 years with blood pressure ≥140/ 90 mm Hg and ≥3 different antihypertensives,  •And/or diabetes for ≥10 years |
| [**Surabenjawong, Thunpiphat [23]**](#_ENREF_23) | Thailand | Single centre, retrospective descriptive study | •Spironolactone  •ACEIs •ARBs | 534 (44)^ | 1 year | Patients taking combination of spironolactone and ACEIs or ARBs■ | NR |

*Data presented as the total number of patients included (total number of patients treated with MRAs), unless otherwise indicated; **Owing to the low number of patients receiving eplerenone, only data from patients receiving spironolactone are reported as MRA users; *** Only part of the patients included were treated with aldosterone antagonists; † Median (IQR); ‡ Mean (SD); ^ Total (CKD); ■ Only relevant subgroups of patients (with CKD) were included into SLR

ACEIs – angiotensin-converting-enzyme inhibitors; ADHF – acute decompensated heart failure; AMI – acute myocardial infarction; ARBs – angiotensin receptor blockers; CKD – chronic kidney disease; CKD -EPI - chronic kidney disease epidemiology collaboration; eGFR – estimated glomerular rate; FU – follow-up; GFR – glomerular filtration rate; HF – heart failure; HFrEF - heart failure with reduced ejection fraction; LVEF – left ventricular ejection fraction; MI – myocardial infarction; MRAs – mineralocorticoid receptor antagonists; NR – not reported; PY – person-years; RAAS – renin–angiotensin–aldosterone system; T2D – type 2 diabetes; UK – United Kingdom; USA – United States of America
